# Distance Motor Learning during the COVID-19 Induced Confinement: Video Feedback with a Pedagogical Activity Improves the Snatch Technique in Young Athletes

**DOI:** 10.3390/ijerph18063069

**Published:** 2021-03-16

**Authors:** Mohamed Abdelkader Souissi, Achraf Ammar, Omar Trabelsi, Jordan M. Glenn, Omar Boukhris, Khaled Trabelsi, Bassem Bouaziz, Piotr Zmijewski, Hichem Souissi, Anis Ben Chikha, Tarak Driss, Hamdi Chtourou, Anita Hoekelmann, Nizar Souissi

**Affiliations:** 1 Research Unit, “Physical Activity, Sport and Health”, UR18JS01, National Observatory of Sport, Tunis 1003, Tunisia; gaddoursouissi@yahoo.com (M.A.S.); omarboukhris24@yahoo.com (O.B.); hichemsouissi82@gmail.com (H.S.); benchikhaanis@yahoo.fr (A.B.C.); h_chtourou@yahoo.fr (H.C.); n_souissi@yahoo.fr (N.S.); 2 Higher Institute of Education and Continuous Training, Virtual University, Montplaisir 2019, Tunisia; 3 Institute of Sport Science, Otto-von-Guericke-University Magdeburg, 39106 Magdeburg, Germany; anita.hoekelmann@ovgu.de; 4 Interdisciplinary Laboratory in Neurosciences, Physiology and Psychology: Physical Activity, Health andLearning (LINP2), UFR STAPS, UPL, Paris Nanterre University, 92000 Nanterre, France; tarak.driss@parisnanterre.fr; 5 High Institute of Sport and Physical Education, Sfax University, Sfax 3000, Tunisia; trabelsi.omar@issepsf.u-sfax.tn (O.T.); trabelsikhaled@gmail.com (K.T.); 6 Department of Health, Exercise Science Research Center Human Performance and Recreation, University of Arkansas, Fayetteville, AR 72701, USA; jordan@neurotrack.com; 7 Research Laboratory: Education, Motricité, Sport Et Santé, EM2S, LR19JS01, High Institute of Sport and Physical Education of Sfax, University of Sfax, Sfax 3000, Tunisia; 8 Multimedia Information Systems and Advanced Computing Laboratory (MIRACL), University of Sfax, Sfax 3021, Tunisia; bassem.bouaziz@isims.usf.tn; 9 Jozef Pilsudski University of Physical Education in Warsaw, 00-809 Warsaw, Poland; piotr.zmijewski@insp.waw.pl; 10 High Institute of Sport and Physical Education Ksar-Said, Manouba University, Manouba 2010, Tunisia

**Keywords:** pandemics, home confinement, weightlifting, motor learning, error detection, kinematic, execution time, biomechanics, feedback

## Abstract

The purpose of the present study was to investigate which of two strategies, Video Feedback with Pedagogical Activity (VF-PA) or Video Feedback (VF), would be more beneficial for the remote error correction of the snatch weightlifting technique during the confinement period. Thirty-five school aged children with at least three months of weightlifting experience were randomized to one of three training conditions: VF-PA, VF or the Control group (CONT). Subjects underwent test sessions one week before (T0) and one day after (T1) a six-session training period and a retention test session a week later (T2). During each test session, the Kinovea version 0.8.15 software measured the kinematic parameters of the snatch performance. Following distance learning sessions (T1), the VF-PA improved various kinematic parameters (i.e., barbell horizontal displacements, maximum height, looping and symmetry) compared with T0 (*p* < 0.5; Cohen’s d = 0.58–1.1). Most of these improvements were maintained during the retention test (T2) (*p*<0.01, Cohen’s d = 1.2–1.3) when compared withT0. However, the VF group improved only twoparameters (i.e., barbell symmetry and horizontal displacement) at T1 (*p* < 0.05; Cohen’s d = 0.9), which were not maintained at T2. Better horizontal displacement and looping values were registered during the retention test in the VF-PA group compared with theCONT group (*p* < 0.05, Cohen’s d = 1.49–1.52). The present findings suggest combining video feedback with pedagogical activity during the pandemic induced online coaching or physical education to improve movement learning in school aged children.

## 1. Introduction

The COVID-19 pandemic has affected educational and sports systems worldwide, leading to the near-total closure of schools, universities and collegesand, consequently, the closure of affiliated training centers for young athletes as a measure to curtail the spread of the infectious disease [1,2,3]. Meanwhile, this global health crisis is triggering the application of distance learning modalities in various fields [4], requiring educators to investigate alternative methods with which to enhance knowledge acquisition in these remote environments [5].

Under normal circumstances, the use of new technologies can more effectively contribute to the improvement of motor learning of sports gestures compared withtraditional approaches. On the one hand, the expert model observation is an important strategy for learning new motor skills in physical education [6,7]. Observation enables the learner to use the movement of the expert model as a reference that would help enforce the reproduction of the movement during the next trials. This reference transmits key information on the effect of the movement and on the movement of the different body segments [8]. In fact, previous studies have argued that video demonstrations are more effective thanthe live versions for the early acquisition of the Kube-Nagi technique [9] and the static pictures used for learning the Ippon-Seoi-Nage in judo [10]. This may be due to the simplification of the visual information in the video-based content in addition to the dual-dimension of this technology. Contrarywise, video feedback is another behavioral procedure used to correctthe skill execution. Video feedback is about showing a learner a video clip of his/her own performance of a particular skill [11]. Moreover, previous studies have shown that, compared withconventional verbal feedback approaches, video feedback interventions during the learning phase of complex skills can result in higher improvements in skill performance [12,13]. One possible explanation for these improvements is that the observation of video feedback allows learners to self-monitor and self-correct the errors detected during task performance. However, others have reported that learners are not capable of grasping all of the relevant information simply from watching video feedback [14]. Indeed, Souissi et al. [8] argued that simply providing a learner with verbal feedback without video feedback or a video feedback without additional cues has little effect on skill acquisition. Therefore, the effects of video and verbal feedback appear to be additive. Recent research in motor learning showed that the active participation of the learner in the learning process improves performance [15,16].

Furthermore, it has been revealed that allowing learners to control the delivery of video feedback can increase self-efficacy [16,17,18], technical performance [16,19], task recall [20] and intrinsic motivation [17,21]. According to Barros et al. [15], limiting how much feedback could be requested seems to increase attention to decisions about feedback and its use. This limitation of requested feedback can cause improvements in performance during retention and transfer tests [22].

Other studies have focused on strategies for using educational videos in teaching and their effects on the understanding of students of the displayed content. Such strategies include segmenting the video into smaller units [23], controlling the pace of the presentation [24] and introducing a pedagogical activity in the form of questions on the latest viewed part of the content while the video is paused [25]. These strategies appear to have a positive impact on learning compared with the continuous viewing of educational videos. They also seem to contribute to a decrease in cognitive load while viewing videos [26,27] and to a smoother cognitive processing [24].

In response to the current situation of quarantine and social distancing, technological advancements are certainly urging sports pedagogists and physical educators to adjust to new methods for providing movement-related feedback [2,28] particularly with regard to new distance learning methods involving video-mediated feedback techniques.

Previous studies have shown that the performance and technique of sports movements decrease after a period of physical inactivity [8,29,30] similar to that experienced during the pandemic. With this in mind, identifying optimal methods of promoting error correction of movements associated with intricate physical activities (i.e., weightlifting, Souissi et al. [8]) via distance learning modalities has become mandatory under such circumstances.

Thus, the aim of the present study was to examine the effects of two augmented feedback strategies, in the absence of the teacher’s intervention, on the correction of technical errors of the snatch during the confinement period and to assess the persistence of the improvements in student learning after a one-week rest period. It was hypothesized that the video feedback visualization method with pedagogical activity could be more suitable for correcting snatch technical errors during the confinement period.

## 2. Materials and Methods

### 2.1. Participants

A total of 35 school aged children (12 females and 23 males) volunteered to participate in the present study. All participants had at least three months of experience of weightlifting in a training center. Parents provided written informed consent for the participation of their children prior to their inclusion in the study. In addition, the study was conducted according to the guidelines of the 2013 Helsinki Declaration. The experimental protocol was approved by the local Research Ethics Committee (CPP: N°0126/2020).

The study sample was selected on the basis of the following inclusion criteria: (i) the ages of the participants was between 10 and 12 years old, (ii) they must have no impaired motor skills that could affect the performance of the snatch task, (iii) they must not be suffering from visual or cognitive problems and (iv) they must own two smartphones, a computer and an internet connection.

After the pretest, participants were randomized into the Video Feedback with Pedagogical Activity (VF-PA = 12), Video Feedback (VF = 12) and Control (CONT = 11) groups. The descriptive characteristics of the participants are further detailed in Table 1.

### 2.2. Procedure

Participants underwent test sessions one week before (T0) and one day after (T1) a six-session training period and a retention test session a week later (T2). The test sessions were focused on the kinematic evaluation of the snatch movement technique. As suggested by Ammar et al. [31,32], all test sessions were performed in the afternoon between 14:00 h and 16:00 h. For all test sessions, participants were asked to maintain normal sleep patterns [33] and not to ingest food at least 4 h before their passage [34,35]. Additionally, participants were asked to avoid caffeine and other caffeinated products (e.g., chocolate, caffeinated gums, caffeine containing beverages) for 48 h before test sessions [36]. The subjects from each group completed three weightlifting training sessions per week for two weeks. Each learning session was comprised of two blocks of twelve repetitions.

Prior to the pretest, participants responded to a questionnaire developed to obtain general information about the availability of the necessary technological equipment (computers and smartphones) at their homes. The different test sessions were conducted under a strict COVID-19 health protocol in accordance with the standards of the World Health Organization.

A week before the experiment, the pretest session was administered and all participants were randomized into three homogeneous groups. Before initiating the learning activities, the following steps were taken: (i) Kinovea software (version 0.8.15) was installed on the computer of each participant of the two experimental groups and (ii) the Zoom mobile application and a videotaped demonstration from an expert were installed on the smartphone of each participant of the three study groups. Three days before the start of the experiment, subjects were familiarized with the general environment, equipment and experimental procedures in order to minimize the learning effect during the study. Participants from all groups performed two distance learning sessions of the forward roll under the supervision of the teacher in order to assess their technological skills. All participants demonstrated a strong ability to use the different technological aids utilized in the present study. A day before the first distance learning session, all participants were equipped with a 5 kg bar and two 21 cm high supports delivered to their homes. Throughout the distance learning sessions, participants of each group maintained their assigned training modes.

#### 2.2.1. Test Procedures

##### Technical Performance

**Figure 1:**
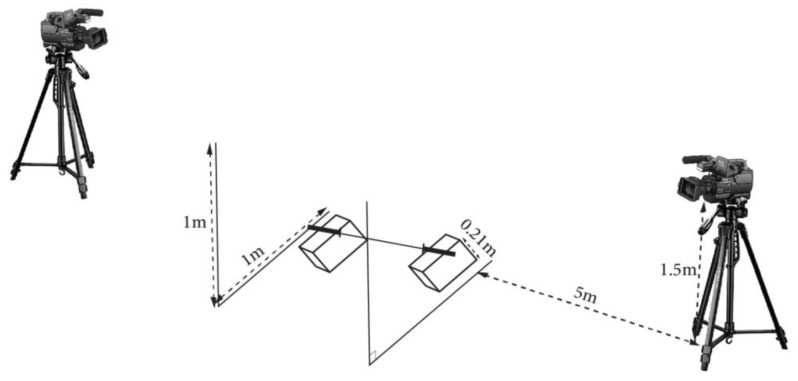
Placement of the cameras during the test sessions.

Each participant had to perform the snatch task using a 5 kg bar placed on two supports at a height of 21 cm in a weightlifting hall. Two landmarks were placed on the vertical plane for each end of the barbell allowing the conversion of the displacement measurements in centimeters. Two digital cameras ((Sony HXR-MC2500, Tokyo, Japan) HD: 50 frames per second (50 Hz frequency)) were fixed on each of the side planes at a distance of 5 m and elevated at 1.5 m from the ground (Figure 1). One camera was on the right and the other one was on the left side of the participant. Two markers were placed on the extremities of the bar.

**Figure 2:**
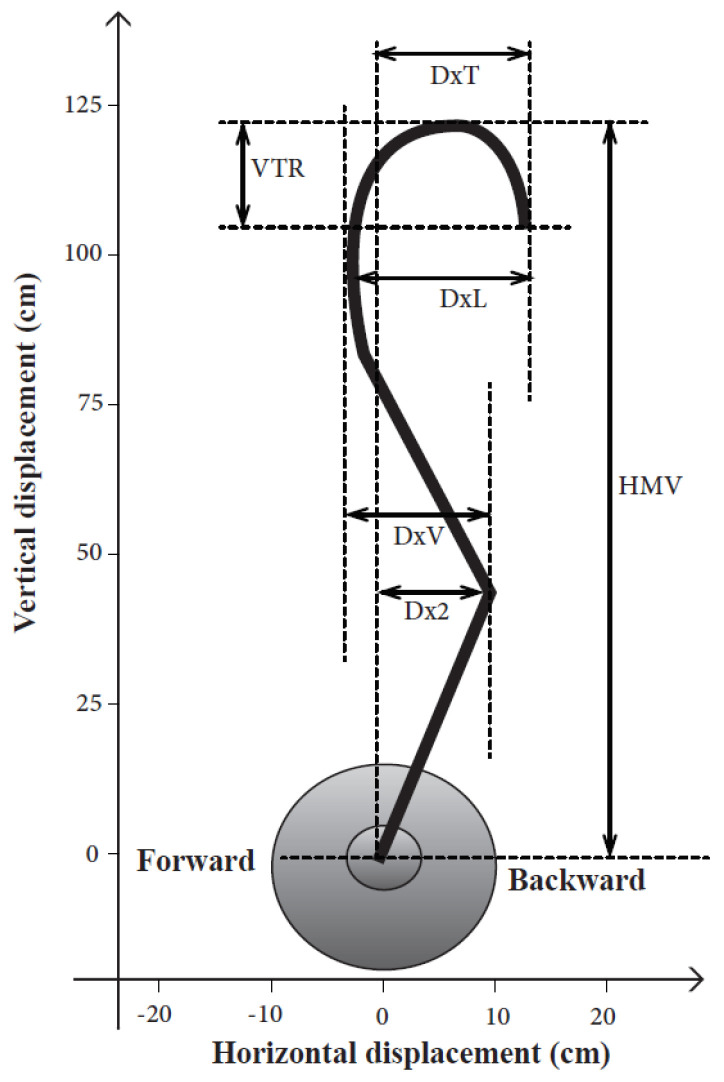
Description of the bar path kinematic variables used to assess quantitative changes in bar positions. Dx2: the horizontal displacement from the start position to the start of the second pull; DxV: the horizontal displacement from the second pull position to the forward position; DxT: the horizontal displacement from the start position to the catch position; DxL: the horizontal displacement from the most forward position to the catch position; VTR: the vertical displacement from the maximum height to the catch position; HMV: the maximum height reached by the bar; Diff Tr: the difference between the left and the right side distances of the bar trajectory in an absolute value.

The collected video sequences were treated by the Kinovea software to provide the horizontal and vertical displacement of the bar. Hoover et al. [37] established a number of important kinematic factors that contribute to a successful snatch technique such as the horizontal (rearward) displacement of the bar in the first pull with respect to the starting position (Dx2), the amount of looping of the bar in the catch phase (DxL), the horizontal displacement of the bar between the receiving position and the reference line (DxT), the horizontal displacement of the bar between the first and the second pulls (DxV), the maximum height reached by the bar (HMV), the maximal vertical displacement of the bar at catch position (VTR) and the difference between the left and the right side distances of the bar trajectory in an absolute value (Diff Tr)(Figure 2).

During the snatch, the trajectory of the barbell is usually an S shaped pattern: the bar must be moved toward the lifter during the first pull and transition phase and then it is pushed away from the lifter’s body by simultaneous hip and shoulder flexions and knee joint extensions [38,39].

Several recent studies have reported the ideal bar path for a proper weightlifting technique with particular attention on reducing the horizontal displacement and the VTR while increasing the HMV. The improvement of the latter parameters allows an increase in power associated with a reduction in energy loss [8,40]. The errors can be classified according to their effect on the outcome of the movement [39].

##### Execution Time

The execution time was defined as the time between the moment at which the bar left the support and the moment at which the bar was stabilized at the end of the reception phase.

#### 2.2.2. Training Session

At the beginning of each distance training session, participants were asked to perform a 15 min self-selected warm-up that included rope exercises, stretching exercises and squat and pull movements.

During the practice, at the beginning of each 12-trial series, participants were asked to view the video demonstration of the snatch skill along with a detailed description of the success criteria (i.e., using audio-visual contents). When the visualization was complete, participants were required to complete 12 repetitions of the demonstrated skill. For both experimental groups, all of the snatch trials were recorded during each training session using a smartphone placed on a chair at a distance of 3 m. In addition, those participants were informed that they would be allowed to get augmented feedback only three times, intercepting the 12 skill trials; no more and no less. The participants of each group received the following feedback modes:

Video Feedback with Pedagogical Activity group (VF-PA): participants in this group were required to watch their video feedback in slow-motion mode. Replay and pause options were available for the convenience of the learner. While watching, each learner had to concentrate on the video content in an attempt to identify errors committed during the skill performance. Learners were provided with key images of the snatch technique including visual cues presented on a paper in order to help them in the error detection and the feedback provision processes. Following the comparison of the video feedback with the paper-based images, learners had to correct the detected errors while performing the next trial.

Video Feedback group (VF): similarly, participants in this group were instructed to watch their video feedback in slow-motion mode with the availability of replay and pause options throughout viewing time in order to detect and correct errors committed during the performance of the snatch. However, this time no pedagogical aids were introduced to assist the learner in the error detection.

Control group (CONT): participants in this group practiced the snatch exercise and did not receive any type of augmented feedback or pedagogical activity.

All distance learning sessions were controlled by the physical education teacher through the Zoom video telephony application. In addition, the parents of participants reported that their children did not practice any snatch exercises outside of the learning sessions.

### 2.3. Statistical Analyses

All statistical analyses were performed using Statistica 10 software (StatSoft, Cracow, Poland). Data are presented as means and standard deviation (mean ± SD). G*power software was used to calculate the required sample size. Values for α were set at 0.05 and power at 0.95. Based on Souissi et al. [8] as well as discussions between the authors, the effect size was estimated to be 0.63. The required sample size was set at 12 participants for each group. The normality of distribution was checked using the Shapiro–Wilks test.

The measured variables were analyzed using a two-way analysis of variance (ANOVA) (three groups (VF-PA vs. VF vs. CONT) × 3 test times (T0, T1 and T2)) with repeated measures for the last factor. The effect sizes were calculated as partial eta-squared (ηp2) to estimate the meaningfulness of significant findings. When appropriate, the significant differences between means were tested using the Bonferroni post-hoc test and the effect sizes were calculated as Cohen’s d [41]. The significance level was set at *p* < 0.05 for all analyses.

## 3. Results

### 3.1. Participant Characteristics

**Figure 3:**
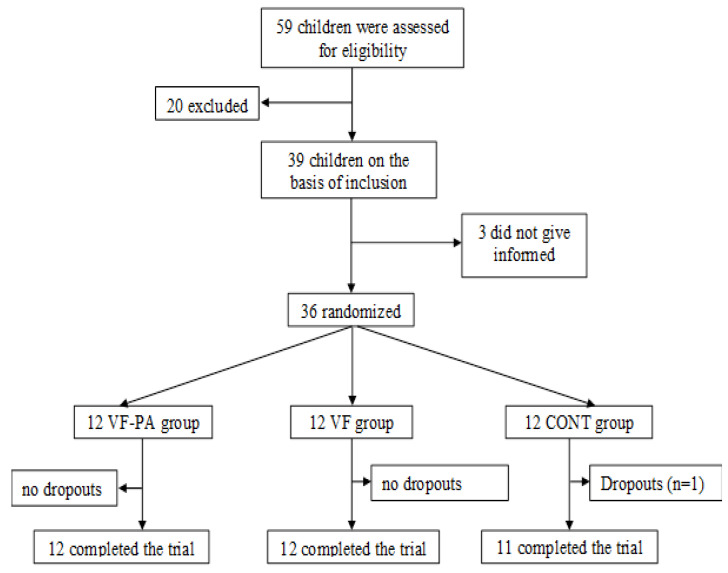
Flow diagram of the study.

Initially, 59 children were identified as potential participants for the present study. However, 20 were later excluded as they failed to meet the inclusion criteria. Three others were as well ruled out due to issues related to signing an informed consent form, which was one of the stipulations for taking part in this study. One child also withdrew prior to the completion of the experiments (Figure 3).

Data of 35 participants (age = 11.06 ± 0.69 years; body height = 146.71 ± 6.19 cm, body mass = 41.47 ± 6.52 kg) were included in the final analysis. Descriptive statistics presented as mean ± SD are summarized in Table 1. For baseline data (T0), a single-factor ANOVA revealed no significant difference between the VF-PA, VF and CONT groups for height, weight, age (Table 1) and all kinematic variables (Table 2) (*p* > 0.05).

### 3.2. Changes in Kinematics Parameters

A mixed-design 3 × 3 (group × time) ANOVA (Table 2) with a repeated measurement of the second factor showed (i) a significant time effect for all kinematic variables with *p* < 0.001 and the ηp^2^ range from 0.21 to 0.26 for Dx2, DxV and Diff Tr with *p* < 0.01 and the ηp^2^ range from 0.14 to 0.17 for DxT, DxL and VTR and with *p* < 0.05 and ηp^2^ = 0.11 for HMV; (ii) a significant group effect for DxL and DxT with *p* = 0.002 and ηp^2^ = 0.21 and 0.22, respectively and (iii) a significant effect of a group-by-time interaction for DxL with *p* = 0.04 and ηp^2^ = 0.14.

Compared withT0, lower values of DxV (21.84 ± 30.47%), DxL (20.20 ± 24.49%), Diff Tr (27.09 ± 58.76%) and HMV (8.11 ± 6.88) were registered for the VF-PA group at T1 with *p* < 0.05 and Cohen’s d range between 0.58 and 1.1. For this group, lower values of Dx2 (27.80 ± 27.11%), DxT (30.59 ± 18.36%) and DxL (22.78 ± 14.77%) were also registered at T2 compared withT0 with *p* < 0.01 and Cohen’s d range between 1.2 and 1.3.

Regarding the VF and CONT groups, the statistical analysis showed a significant difference only between T1 and T0 among the VF group for DxV (22.97 ± 24.68%) and Diff-Tr (32.96 ± 30.52%) with lower values at T1 (*p* < 0.05 and Cohen’s d = 0.9).

No significant differences were found between T1 and T2 in all groups.

Additionally, the post-hoc test showed no significant differences between groups after the training intervention (T1) and only a significant inter-group difference between the VF-PA and CONT groups at T2 for the DxT and DxL parameters (*p* < 0.05, Cohen’s d = 1.49–1.52).

### 3.3. Execution Time

**Figure 4:**
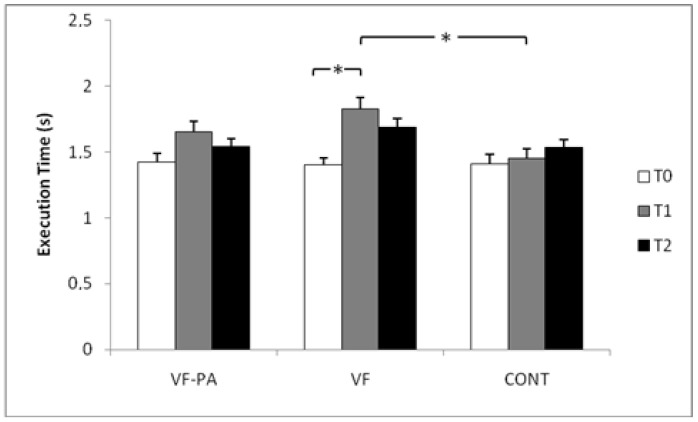
Execution time (mean ± standard deviation) at T0, T1 and T2 among the three groups. VF-PA: Video Feedback with Pedagogical Activity group; VF: Video Feedback group; CONT: Control group. * Significant difference at *p* < 0.01.

The execution time at T0, T1 and T2 for all groups are presented in Figure 4. The two-way ANOVA showed significant effects of time (F = 9.17, *p* < 0.001, ηp2 = 0.22) and group (F = 4.83, *p* < 0.05, ηp^2^ = 0.23).

Compared with T0, a higher execution time (36.11 ± 40.49%) was registered only for the VT group at T1 (*p* < 0.05, Cohen’s d = 1.53). Additionally, the post-hoc test showed significant higher values for the VF group compared with the CONT group at T1 (*p* < 0.01, Cohen’s d = 1.44).

## 4. Discussion and Conclusions

In the present study, different strategies of remote correction of technical errors in weightlifting during the confinement period were employed to investigate which would have the greatest effect on improving the performance. Certain kinematic parameters such as the horizontal and vertical displacement of the bar and the difference between the left and the right side distances of the bar trajectory [8,39] were measured to quantify changes resulting from the different learning conditions. After six distance learning sessions, the results supported the assumption that VF-PA (compared with VF) may be more beneficial for the snatch technical performance with regard to the barbell trajectory pattern; this benefit also remained present in the retention test. The results of the comparisons between participants of the different study groups showed that the technical performance of the VF-PA group was better for DxT and DxL kinematic variables at T2 compared with the CONT group.

The findings of the present study revealed greater decreases at T2 in the VF-PA group for the horizontal displacement from the start position to the beginning of the second pull Dx2 (−28%) and from the horizontal displacement of the bar between the receiving position and the reference line DxT (−31%). The obtained results were similar to those of Kok et al. [16] who confirmed a positive effect of video feedback combined with visual cues on learning of the shot-put technique in physical education. This significant improvement did not appear at T1 due to a sudden change in the practice environment. However, through practicing VF-PA, it could be expected that improvements might emerge with time. A possible reason for these improvements at T2 might be the effect of the focus of attention. During the post-learning session, the automatic control process was likely to be disrupted by the VF-PA strategy, causing a reduced technical performance. However, a week after the retention test in the absence of feedback, the automatic control process was likely to take priority. Feedback may also be assimilated more slowly through VF-PA as the process involves conscious reasoning. This suggests why learning effects might take longer to appear.

Moreover, the results demonstrated that the performances of participants from the VF-PA group improved at T1 in terms of the kinematics displacement of the bar between the first and the second pulls DxV (−22%), the difference between the left and the right side distances of the bar trajectory Diff-Tr (−27%), the maximum height reached by the bar HMV (8%) and the amount of looping of the bar in the catch phase DxL (−20%). These improvements in the DxL parameters persisted at T2 (−23%). Several previous studies [8,39] have shown that reducing the horizontal displacement of the bar improves the snatch technique and decreases the risk of injury. The findings of the present study were consistent with those of Cheon et al. [25], showing that the integration of a pedagogical activity (in the form of questions) during the visualization of educational videos improved performance. In addition, previous studies have demonstrated that the integration of different forms of attentional guidance during video visualization improves learning of the soccer pass [42] and the weightlifting technique [43] in adult athletes. One possible explanation for this improvement could be that the introduced pedagogical activity directs the attention of the learner to make logical comparisons in key phases of the movement. During these comparisons, the learner exerts efforts to detect the errors committed during the skill execution and tries to correct them in the subsequent trials. The VF-PA strategy provides the learner with new intrinsic feedback, stimulating the functions of perceptive categorization and the conceptual and symbolic elaboration of the received information. In addition, encoding and retrieving information during embedded pedagogical activity may enhance the schema construction of novices and produce a higher performance [25].

Concerning the VF group, the results of the present study showed a significant decrease for DxV and Diff-Tr at T1 (−23% and −33%, respectively). These findings were similar to the results of Potdevin et al. [44] who declared that video feedback improved gymnastic skill technical execution during physical education lessons at school. A possible reason for this improvement might be the effect of the active participation of the learner in the learning process (a choice of when to watch the feedback video). In addition, the total control of the video visualization using the pause and/or replay buttons can improve the rate of detection and correction of errors committed during complex movements. Another possible reason for this improvement may be due to further information processing during the execution of the snatch in the post-test. The learner tries not to repeat detected errors during the acquisition phase. In fact, this slowing seems to be primarily the result of a strategically conscious control to prevent execution errors [45]. In increasing the execution time of movement, the central nervous system becomes able to make technical adjustments by means of simultaneous internal feedbacks, specially through basal ganglia [46] and cerebellum [47] corrections. Regarding the Dx2, DxL, DxT and VTR parameters, the findings of the present study showed no change (*p* > 0.05). These findings werein agreement with the study of Souissi et al. [8], which suggested that this pattern could be explained by the fact that beginners are not capable of grasping all relevant information from the total amount of information displayed in the video feedback.

To our knowledge, this is the first study to investigate distance motor learning in sports settings during the confinement period. This study aimed to compare the effectiveness of two technique error correction strategies in a complex weightlifting skill that depended on several degrees of freedom. However, a possible limitation of the study could be that the video feedback viewing time and the maximum speed and height of the bar were not measured.

The present findings suggest combining video feedback with pedagogical activity during pandemic induced online coaching or physical education to improve movement learning in school aged children. The obtained results of the VF-PA strategy might influence future methods of distance coaching and training and can also be considered for research in different fields such as physical education, physiotherapy and coaching.

## Figures and Tables

**Table 1:** Descriptive characteristics (mean ± standard deviation) of the participants.

Groups	VF-PA (*n* = 12)	VF (*n* = 12)	CONT (*n* = 11)
Age (years)	11.06 ± 0.74	11.10 ± 0.71	11.03 ± 0.65
Height(cm)	146.71 ± 6.33	146.92 ± 5.95	146.45 ± 6.85
Body mass (kg)	40.29 ± 5.86	41.73 ± 5.54	40.41 ± 6.52
Body mass index (kg/m^2^)	19.08 ± 1.36	19.24 ± 1.26	19.17 ± 1.43

VF-PA: Video Feedback with Pedagogical Activity; VF: Video Feedback.

**Table 2:** Kinematic parameters (mean ± standard deviation) at T0, T1 and T2 among the three tested groups.

Parameter	VF-PA			VF			CONT			ANOVA		
	T0	T1	T2	T0	T1	T2	T0	T1	T2	Time × Group	Time	Group
**Dx2 (cm)**	11.40 ± 3.3	8.65 ± 3.15	7.72 ± 2.8 **	12.11 ± 3.07	10.19 ± 3.61	10.4 ± 3.07	11.82 ± 3.3	11.09 ± 3.02	11.19 ± 3.44	*p* = 0.22	F = 8.47; *p* < 0.001; ηp^2^ = 0.21	*p* = 0.14
**DxV (cm)**	14.63 ± 3.53	10.87 ± 3.38 *	11.77 ± 3.16	14.33 ± 3.21	10.94 ± 3.99 *	12.4 ± 3.69	14.54 ± 3.06	14.17 ± 3.3	14.19 ± 3.27	*p* = 0.15	F=9.61; *p* < 0.001; ηp^2^ = 0.23	*p* = 0.21
**DxT (cm)**	19.5 ± 5.35	15.26 ± 5.02	13.18 ± 4.28 ** #	21.21 ± 6.25	18.95 ± 4.74	17.84 ± 2.4	20.14 ± 5.49	20.35 ± 4.11	20.9 ± 5.74	*p* = 0.052	F = 5.48; *p* = 0.006; ηp^2^ = 0.14	F = 4.42; *p* = 0.002; ηp^2^ = 0.22
**DxL (cm)**	22.74 ± 5.17	17.47 ± 5.05 *	17.23 ± 4.18*#	23.43 ± 5.0	19.69 ± 4.86	19.84 ± 4.18	22.86 ± 4.61	23.44 ± 4.46	23.91 ± 4.77	F = 2.62; *p* = 0.04; ηp^2^ = 0.14	F = 5.9; *p* = 0.004; ηp^2^ = 0.16	F = 4.25; *p* = 0.002; ηp^2^ = 0.21
**VTR (cm)**	17.03 ± 3.42	14.47 ± 4.71	14.49 ± 4.16	16.63 ± 4.17	13.12 ± 3.18	14.12 ± 3.82	18.19 ± 4.79	16.88 ± 4.75	17.09 ± 4.25	*p* = 0.78	F = 6.41; *p* = 0.003; ηp^2^ = 0.17	*p* = 0.13
**HMV (cm)**	130.54 ± 18.08	140.46 ± 16.1 *	138.37 ± 13.28	129.38 ± 16.72	132.92 ± 18.13	132.79 ± 16.53	129.62 ± 15.44	128.09 ± 18.08	130.65 ± 18.82	*p* = 0.11	F = 3.89; *p* < 0.05; ηp^2^ = 0.11	*p* = 0.56
**Diff-Tr (cm)**	14.1 ± 7.04	9.04 ± 5.06 *	9.78 ± 6.2	14.82 ± 5.99	9.62 ± 4.63 *	11.2 ± 4.84	14.65 ± 6.07	13.51 ± 5.35	13.74 ± 6.54	*p* = 0.24	F = 11.51; *p* < 0.001; ηp^2^ = 0.26	*p* = 0.36

VF-PA: Video Feedback with Pedagogical Activity group; VF: Video Feedback group; CONT: Control group; Dx2: the horizontal displacement from the start position to the start of the second pull; DxV: the horizontal displacement from the second pull position to the forward position; DxT: the horizontal displacement from the start position to the catch position; DxL: the horizontal displacement from the most forward position to the catch position; VTR: the vertical displacement from the maximum height to the catch position; HMV: the maximum height reached by the bar; Diff Tr: the difference between the left and the right side distances of the bar trajectory in an absolute value; *p* values were adjusted for multiple testing by the Holm–Bonferroni method.*, ** Significant difference compared with T0 (*p* < 0.05 and *p* < 0.01, respectively).# Significant difference compared with CONT (*p* < 0.05).

## Data Availability

Data are available from the authors (M.A.S., or A.A.) upon reasonable request.
